# Trees Wanted—Dead or Alive! Host Selection and Population Dynamics in Tree-Killing Bark Beetles

**DOI:** 10.1371/journal.pone.0018274

**Published:** 2011-05-25

**Authors:** Kyrre L. Kausrud, Jean-Claude Grégoire, Olav Skarpaas, Nadir Erbilgin, Marius Gilbert, Bjørn Økland, Nils Chr. Stenseth

**Affiliations:** 1 Department of Biology, Centre for Ecological and Evolutionary Synthesis (CEES), University of Oslo, Oslo, Norway; 2 Norwegian Forest and Landscape Institute, Ås, Norway; 3 Lutte Biologique et Ecologie Spatiale, Université Libre de Bruxelles, Bruxelles, Belgium; 4 The Norwegian Institute for Nature Research, Oslo, Norway; 5 Department of Renewable Resources, University of Alberta, Edmonton, Alberta, Canada; 6 Biological Control and Spatial Ecology, Université Libre de Bruxelles, Brussels, Belgium; 7 Fonds National de la Recherche Scientifique, Brussels, Belgium; University of Maribor, Slovenia

## Abstract

Bark beetles (*Coleoptera: Curculionidae*, *Scolytinae*) feed and breed in dead or severely weakened host trees. When their population densities are high, some species aggregate on healthy host trees so that their defences may be exhausted and the inner bark successfully colonized, killing the tree in the process. Here we investigate under what conditions participating with unrelated conspecifics in risky mass attacks on living trees is an adaptive strategy, and what this can tell us about bark beetle outbreak dynamics. We find that the outcome of individual host selection may deviate from the ideal free distribution in a way that facilitates the emergence of tree-killing (aggressive) behavior, and that any heritability on traits governing aggressiveness seems likely to exist in a state of flux or cycles consistent with variability observed in natural populations. This may have implications for how economically and ecologically important species respond to environmental changes in climate and landscape (forest) structure. The population dynamics emerging from individual behavior are complex, capable of switching between “endemic” and “epidemic” regimes spontaneously or following changes in host availability or resistance. Model predictions are compared to empirical observations, and we identify some factors determining the occurrence and self-limitation of epidemics.

## Introduction

Bark beetles have coexisted with their tree hosts since the early Mesozoic [1], and while often regarded as pests, bark beetles and their associated fungi also play important roles in nutrient cycling, forest dynamics and biodiversity [2]–[5]. But of the more than 5800 described bark beetle species, less than a dozen, mostly in the genera *Dendroctonus* and *Ips* (e.g., *Ips typographus*, *Dendroctonus ponderosae*, *D. frontalis*) are known to colonize and kill even healthy host trees [2] when population densities are high [3]. Aggregation pheromones released by beetles while they are boring into and excavating mating galleries in host trees elicit attraction of conspecifics of both sexes, and the greater the number of beetles attacking, the greater their probability of exhausting host defences and achieving successful oviposition [4]. The beetles also vector presumably mutualistic microorganisms (mostly fungi), some of which contribute to tree mortality [2].

The Pinaceae have evolved defences against bark beetles and their associated fungi [5]: resin-filled ducts can mechanically seal off the entrance holes, a number of compounds (terponoids and phenolics) with inhibitory or toxic effects on the beetles and fungi increases in concentration, and cell structure changes in the surrounding tissue helps contain the infection [1]. The effectiveness of these defences varies over time within and between trees, as it is vulnerable to water stress and other biological factors. While a fallen tree with remaining root contact may still have partially active defences, broken trees are defenceless [1], [6] and suppressed trees have reduced defences. As modular organisms, the “death” of a tree is neither instantaneous nor necessarily affecting the whole individual. Here, however, we define a “dead” tree as one that has no effective defense against a given species of bark beetle. This often results from loss of root contact, extreme drought stress, mechanical damage or parasites.

Failure to find a suitable host is a major source of beetle mortality [7], and usable breeding habitat is patchy, stochastic and transient, as dead host trees appear randomly in the landscape through wind-felling or logging, after which the phloem decays within months, while forest succession take decades to centuries. On the other hand, partaking in an unsuccessful aggregation is at best a waste of time and at worst fatal, and the risk is likely to be greatest for the ones initiating the attack. Since the maximum reproductive success may be achieved at low to intermediate gallery densities, the beetles seem to face both positive and negative density dependence, on slightly different spatial scales [12]–[14]. At very low densities, mate finding may be problematic, while increasing densities both cause crowding and facilitates colonization of living trees. Early colonizers of living trees meet the strongest tree defences and uncertain success, latecomers meet higher competition and, perhaps, increased predation [8].

From an evolutionary perspective, this raises several questions: Why do individual beetles initiate or join with unrelated conspecifics in risky “cooperative” attacks? This is especially puzzling considering that the beetles initiating the attack face the greatest risks, and thus are the least likely to reap the benefits. Conversely, if such attacks represent an adaptive strategy, why are they nevertheless only sporadic, usually local and self-limiting, but at other times forming large-scale outbreaks that kill virtually all host trees over large areas, persisting long after the triggering resource pulse has ceased [9], [10], [11], [12]?

Here we develop an individual-based model, hereafter called the Sequential Restricted Distribution (SRD), to study the adaptive strategies of host selection (dead vs. living hosts) that lie behind the dynamics approached at the population level by traditional population models [13]. The model explores what behavior is adaptive (i.e., maximizing fitness, here measured as expected reproductive success) under what circumstances. It then uses the results to explore the population dynamics that emerge from the predicted behavior. Thus, we can approach the evolutionary mechanisms behind the conditional strategies that make some species and populations switch between endemic and epidemic states. (The term “epidemic” is here used to denote all populations that kill living trees. This does not imply that all populations entering the “epidemic” state will produce large-scale tree killing, only that large-scale forest mortality results when populations in a large number of patches become epidemic within a relatively short time due to a combination of dispersal and autocorrelated climate and landscape effects. Also, some obligatory saprophagous species are not necessarily described in this model.)

**Figure 1:**
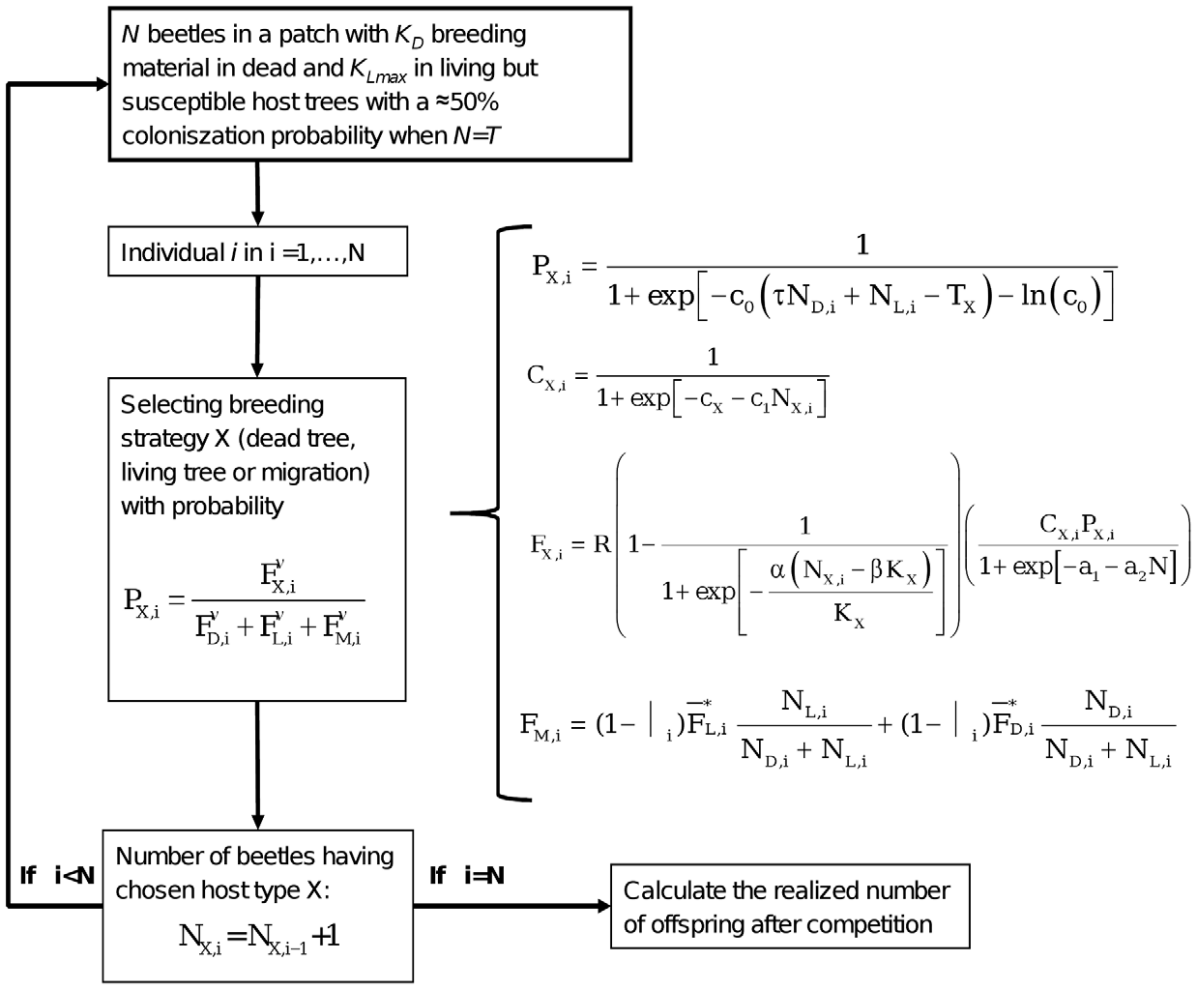
Schematic overview of the model, summarizing the steps and equations.

To do this, we couple a set of monotonic functions (Fig. 1) representing the trade-offs facing the individual beetle: the probability that a living tree will become colonisable (Eq. 1), the risk suffered by the first beetles boring into a still living tree (Eq. 2), the decrease in reproductive success caused by high gallery densities (Eq. 3), and migration risk (Eq. 4). We scale the parameters (see Analysis section belo and Table 1) so that model runs are biologically reasonable, investigate the effect of varying parameters representing biological differences, and calculate to what degree our inclusions of relative risk and sequential choice predict divergence from the Ideal Free Distribution (IFD).

**Table 1:** The model parameters which can be set independently, the interval over which they have been defined or assessed, and their general effect.

	Parameter description		Effect of increasing value over interval
N	Population density	0–100	A number of “individual” beetles representing the whole population that encounter a habitat patch during swarming. The parameter to which everything else is scaled.
K_D_	Dead host tree abundance.	0–25	Allows offspring production by more individuals.
K_L_	Susceptible host tree abundance.	0–25	Allows offspring production by more individuals if are successfully colonised.
T	Colonisation (defence) threshold, giving the N for which 50% of the broods in living host trees are successful when c_0_ = 1	5–50 (100)	Increases the swarm density (N) at which tree colonisation, may happen, and thus the pay-off relative to dead trees and migration over a range of densities. At very high values no trees are colonised and F(N) is unimodal.
τ	Proportional contribution to live tree defence exhaustion by beetles settling in dead trees	0–1	Decreases the population density (N) where living trees are being colonized. Thus, successful colonisations and epidemics are more likely.
c_0_	Per capita contribution to successful colonisation of living hosts.	0.01–1	Increasing the rate of colonisation, and thus the steepness of the colonization threshold, and thus the pay-off from colonisation of living hosts.
α	Per capita contribution to negative density dependence.	0.11–4	Destabilises population dynamics, increasing bimodality of F(N),
ω_1_	Logit-probability of surviving migration to another patch at the start of the swarming season	−2–3	Greater values of Ω (eq.4) decrease the mean proportion of beetles migrating. At low values it prevents living trees from being attacked, increasing values destabilizes epidemic populations by increasing overcrowding.
ω_2_	Give the rate of changing migration mortality with time, with time defined as the number of individuals who have settled.	0–1	
β	Regulate point where negative density dependence start occurring.	0–0.3	Greater values increase the density at which crowding starts reducing reproductive output, affecting population dynamics. Small impact on dynamics, none on distributions.
R	Per capita contribution to the next breeding generation when including density-independent mortality.	1.5–50	Increase population growth rate, destabilising population dynamics and increasing the likelihood of shifting from one dynamical regime to the other.
a_1_	Log-probability of successful mating when N = 1.	−3–3	The swarm density under which reproductive output is decreased due to Allee effects. Impacts low-population dynamics.
a_2_	Steepness of Allee effects	0–3	
c_L_	Logit-probability of successful reproduction when N_X_ = 1, relevant in living trees where tree defences pose risk to early colonisers.	5	Decreases the payoff for early colonisers, and thus the degree of crowdedness and migration mortality under which initiating attacks is an adaptive strategy. When this risk is overcome, the switch to epidemic dynamics is all the more abrupt, especially when c_1_ is high.
c_D_		−3–1	
c_1_	Per capita decrease in risk from being an early coloniser.	0–3	
ν	The precision with which individuals identify the optimal strategy	10–100	Greater values decrease stochasticity. No effect on mean result.

The IFD (conceptually similar to the game theory term Nash Equilibrium) is a central idea in evolution and behavioral ecology [14], [15]. It predicts that organisms should distribute themselves proportionately to the amount of resources available in each patch. The relationship with evolutionary game theory is that when an IFD is achieved, no individual can do better by changing patch. Imperfect information, unequal competitive abilities, time lags and costs of redistribution can inhibit the formation of an IFD, but it is a very useful null assumption, as systematic deviations from it alerts us to the existence of costs and/or constrains that need to be accounted for.

Finally, the results are compared to empirical data and existing population models [13], and discussed in relation to evolutionary, ecological and management issues.

## Analysis

Scaled logistic functions are used as approximations of the actual risk and fitness trade-offs [16], [17], [18], as we can assume monotonic but not linear transitions between the biologically plausible extremes [18]. For instance, a single beetle will never overwhelm a tree but an infinite number of beetles always will, the risks of attacking or migrating are between zero and certain death, and mean number of offspring per adult is between zero and the maximum for the species. Our results are general over a biologically plausible range of parameters (see below), and do not depend on the exact functions or parameter values as long as they are monotonic and scale relatively to each other. The following assumptions are made in the SRD model:

1. **The beetles show an adaptive behavioural reaction norm.**
  Thus, we assume that they have had time to evolve, and that there are no strong evolutionary trade-offs with processes invisible to this model, or manipulation from other organisms such as parasites. Kin-selected altruism is assumed to play no significant part, since at least some important species are outbreeding and widely dispersing [19], [20], [21],
2. **The beetles respond to the density of conspecifics, and to the defence level of their host trees.**
  While uncertain, the beetles' estimates are assumed to be unbiased. This is supported by the observation that beetles respond to host volatiles, conspecific pheromone concentrations and post-landing host inspection [22], [23], [24], [25], [26], [27].
3. **The beetles act sequentially, within a limited time (flight period).**
  Individuals must at some point make a choice of one resource over the other. As they can only be aware of conspecifics that have already settled and started releasing volatiles and pheromones, they have no information about the presence of unsettled individuals or about individuals that will arrive later.

The model proceeds through several steps (Fig. 1). First, beetle no. 1 selects the strategy (settling in a dead tree, settling in a living tree, or migrating away) that gives it the highest expected number of offspring. Then beetle no. 2 does the same, but the outcome may be influenced by the choice already done by beetle no. 1. This is repeated until all N beetles that encounter the patch during a swarming period have settled or migrated. The result of these choices determines the adult (gallery) densities in dead and live trees respectively, and thus the number of realized offspring.

The patch size is an abstraction of the maximum distance over which the beetles integrate information about their hosts and conspecifics using olfactory and, at close range, even visual/tactile clues. From pheromone trapping experiments, we consider that about a hundred meters or less in radius seems a realistic scale approximation [28], [29], and it can reasonably be visualized as a stand of host trees. N is the number of beetles/patch, defined as the number of beetles that will respond to a patch during the flight period. Using the patch (stand) as the spatial unit, N is hence referred to as “population density”.

The amount of breeding material in a patch exists either in the form of dead (i.e., undefended) and living (i.e., defended but susceptible to colonization) trees, which are denoted K_D_ and K_L_ respectively. This is conceptually related to the “carrying capacity”, and is scaled to the density at which mean reproductive success is halved. The subscripts D, L and M are used throughout, denoting dead trees, live trees and migration respectively.

Assuming a probabilistic relationship [17] regulated by c_0_, the expected probability of beetle *i* reproducing successfully if it chooses a living host is a function of tree resistance (T), how many conspecifics have already attacked (N_L_), and possibly also of how many conspecifics have settled in the dead trees (N_D_) in the patch. Since some of these may have first sampled living trees [30], penetrating resin channels, their average contribution towards successful colonization is reduced to a proportion τ, where τ ∈ (0,1). Thus:
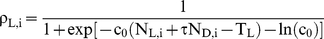
(1a)

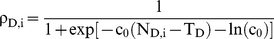
(1b)This is closely related to the number of trees being killed, although some species, such as *D. ponderosae*, can succeed in so-called “strip-kills” where only a section of the tree dies [16].

In addition, the first *i* beetles settling on a tree may suffer an increased risk C_i_ of not rearing offspring due to the still-vigorous tree defences. As more beetles settle, defences are exhausted and the risk faced by subsequent settlers decrease:
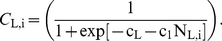
(2)We assume essentially no risk for initial settlers in dead trees (c_D_>3, T_D_<−5/c_0_), but a substantial risk for the first settlers in live trees (c_L_<3, T_L_>5). The number of offspring F produced by the i^th^ beetle can reach its maximum value of R up until the point where competition starts reducing reproduction with increasing gallery density (eq.3). Here α and β regulate the onset and steepness of negative density dependence, and a_1_ and a_2_ regulate a reduced probability of reproduction due to mate finding failure or other Allee effects.
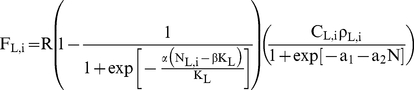
(3a)

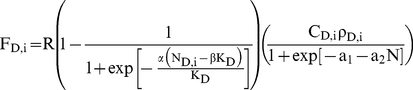
(3b)


The expected reproductive output from out-of patch migration F_M,i_ is the better of the expected (mean) values of (1−Ω_i_)F_D,i_ and (1−Ω_i_)F_D,i_ where Ω is the probability of dying before finding a new patch to settle in. This may increase with time as fat reserves are depleted and time runs out so that
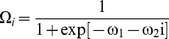
(4)F_M_ assume that K_D_ and K_L_ are mean values of a Poisson-distributed resource landscape, and that N, N_L,i_ and N_D,i_ are mean values with Gamma-distributed values in the receiving patches, thus including a shape parameter to regulate the standard deviance γ. If the population is perfectly synchronized (γ = 0), a migrant will always meet the same N_L_ and N_D_ (though K still varies). As migration range increases relative to the scale of spatial population synchrony (γ increases), the population state experienced by emigrants is increasingly independent of the state they left. Thus, the local model implicitly accounts for the larger-scale process of migration [31].

As each beetle optimizes its expected reproductive success, the probability P that the i^th^ beetle will choose each strategy is
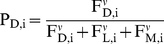
(5a)

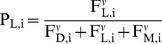
(5b)

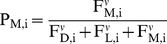
(5c)where 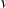 regulates the sensitivity of beetles to differences in fitness between substrates (i.e., imperfect information).

If the reproductive outputs of all beetles in a tree react equally to the final density, the realized number of offspring after the settlement process is complete becomes
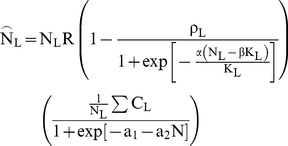
(6a)

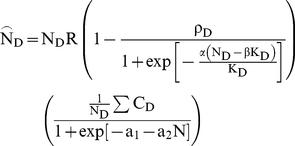
(6b)


**Figure 2:**
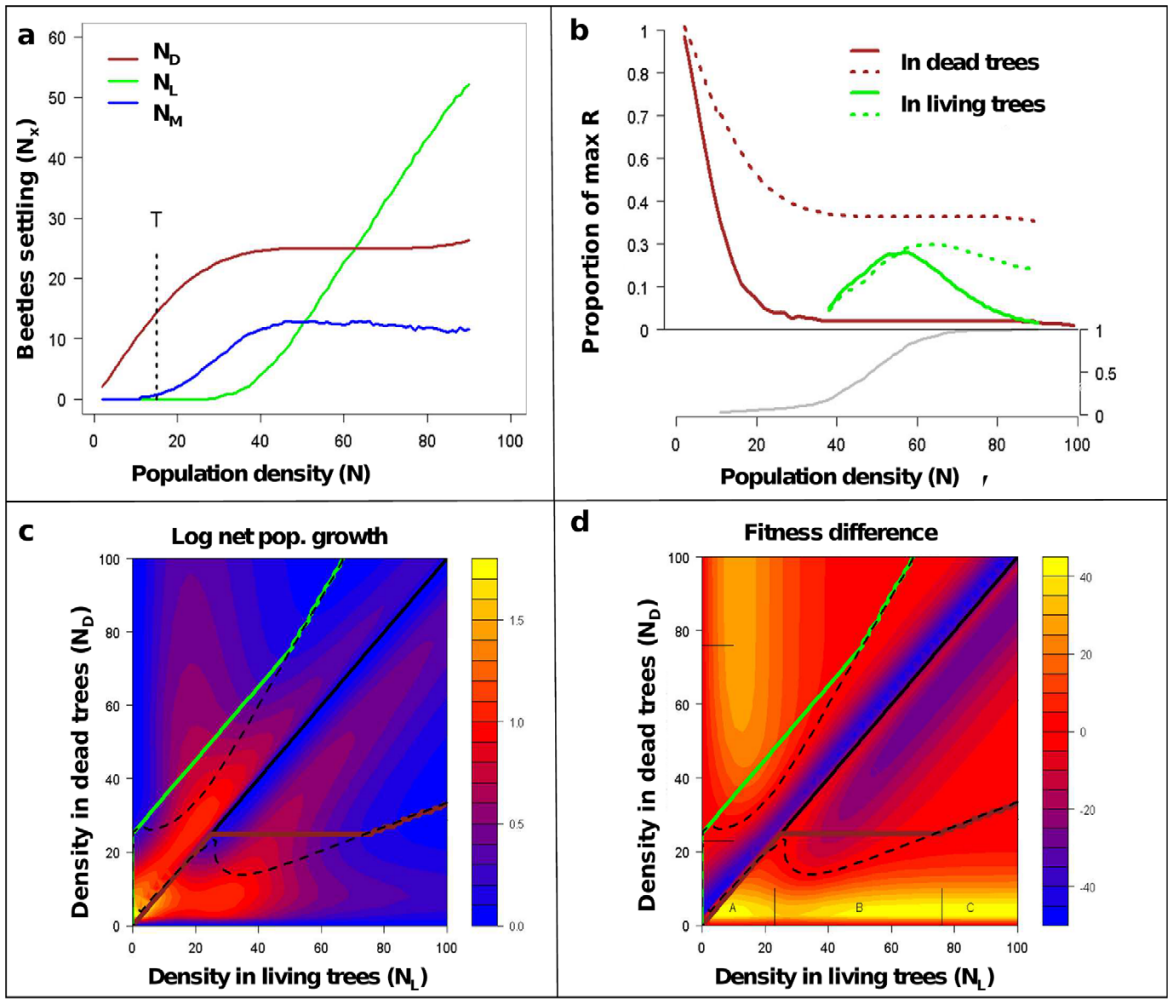
Predictions of the SRD for a set of parameters giving potential tree mortality. a) The density of beetles settling in dead trees (brown), living trees (green) or migrating (blue) in one flight season as functions of swarm density (N). Here K_D_ = 5, K_L_ = 10, median Ω = 0.6, α = 1, c_0_ = 0.2, c_T_ = −2, β = 0.05, c_1_ = 0.5, a_1_ = −1, a_2_ = 5,R = 10, τ = 0.5. The threshold T = 15 is marked and shows where living trees would be colonized with P = 0.5 if all beetles had joined attacks. **b**) The resulting fitness functions (expected number of offspring per capita) when early-arriving individuals are able to monopolize resources (dotted lines) and when they are not solid). The grey line shows the probability of successful colonisation of living trees increasing with population density. **c**) The colours show total population growth rates as a function of beetle distributions, showing the stable distributions as predicted by the IFD (dotted) and SRD (green and brown solid) lines. Below the diagonal, the horizontal axis shows population density(N), the vertical axis the number of beetles settling in dead trees (N_d_). Above the diagonal, the vertical axis shows population density, the horizontal axis the number of beetles settling in living trees (N_s_). **d**) As in (c), except that colours show per cent difference in fitness between beetles in dead and living trees. Following the brown (dead-tree) line, we see that at low densities (interval A) both the SRD and IFD predict all beetles to settle in dead trees. As living trees are settled (interval B) we see marked deviations from the IFD as individuals colonizing living trees enjoy increased fitness. However, as population density increases further, the SRD and IFD converge (interval C) as both resources become crowded.

**Figure 3:**
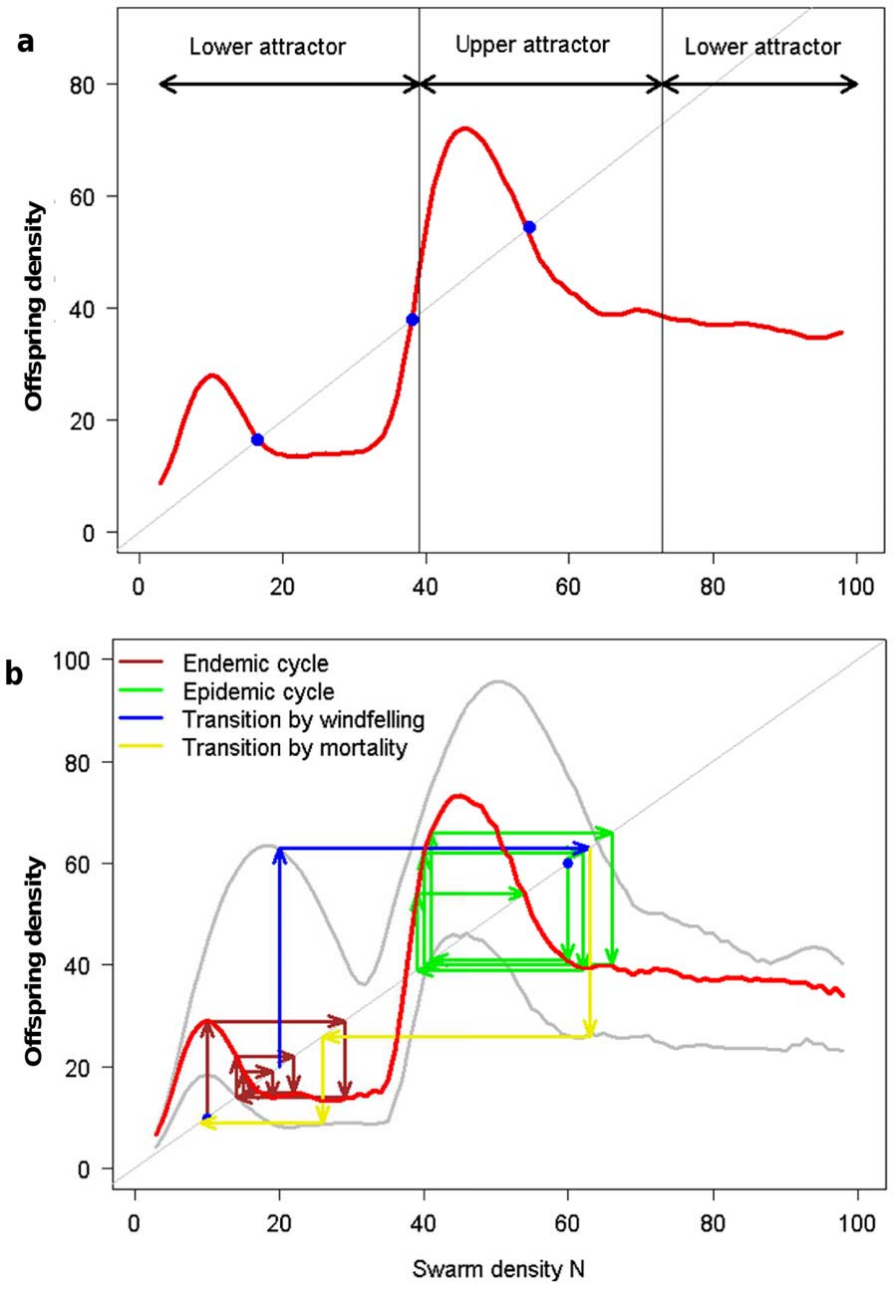
Population dynamics of the SRD. a) Offspring density as a function of swarm density shows three non-zero equilibrium points (blue dots), and the population trajectories have two attractor basins; a lower (endemic) and higher (epidemic). **b**) As (a), showing one endemic (brown arrows) and one epidemic (green arrows) trajectory. A population may be transported from one attractor basin to the other by several mechanisms in either direction (blue and yellow arrows). For instance a large windfelling (giving a brief doubling of K_D_, upper grey line, blue arrows), or a winter of poor survival (a decreased R, lower grey line, yellow arrows).

**Figure 4:**
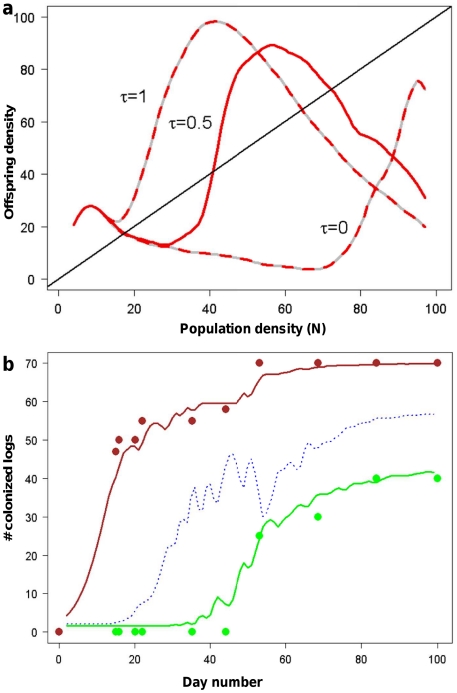
Predictions of the SRD. a) The effect of varying the random sampling coefficient (τ) – i.e., the chance that a beetle bores into a living tree, possibly piercing resin channels and transferring fungi, before settling in a dead tree. We see that beetle species/populations with low a sampling rate are predicted to be less likely to colonize trees, and less likely to sustain continued epidemic states. **b**) The brown points show the number of dead trees (i.e., logs) and green points the number of living trees that were observed to be colonized by *I. typographus* at a site over a period of 100 days (data from Grégoire 1996). The colonization sequence predicted by the model (brown line for beetles settling in dead trees, green for live trees and blue for migration) is highly consistent with these observations (see Analysis and Results sections).

The total number of offspring produced in the patch is thus 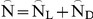 But if each beetle monopolized the amount of bark it can utilize for breeding when it settled, thus suffering no interference from later arrivals, the total number of offspring becomes
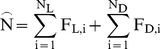
(7)A summary of the SRD is given in Fig. 1. For numerical investigation of the model, we scale K_D_, K_L_, T_L_ and the other parameters (Table 1) so that when 0≤N≤100 most model runs will be biologically reasonable and non-trivial (i.e., populations non-zero and not increasing to infinity, positive “carrying capacities”, live trees not succumbing to one single beetle etc.). We here scale N to the range of 0–100 individuals to make numerical analysis computationally manageable, but running the model upscaled to more realistic population numbers gives the same biological predictions. For an overview of model predictions see Fig. 2a–d, and for the population dynamics following from the model Fig. 3a–b and 4a.

Grégoire et al. [32] describe a site in Southern Belgium with 70 spruce that had been felled by wind in February 1990 and left on site. The number of windfalls and living trees in the same stand that were colonized by *I. typographus* were estimated on 13 occasions from April 10^th^ to November 12^th^. From the asymptotic increase (Fig. 4b), we assume that the number of trees settled at the end of the period represent all that were available in the patch, and that beetles encountered the patch at an approximately constant rate. With dead and live trees of equal size, the data tells us that K_L_/K_D_ = 44/70, and to run the model from 0 to 100 representing the (unknown) number of real beetles that arrived over the period, we scale this with a factor g_0_ = 0.1. We then investigate whether the colonization pattern will be reproduced using plausible values of the within the investigated range for the other, unknown, variables. The proportion of trees observed to be colonized is expected to increase logistically in proportion to the number of individuals settling in them, so that the number of trees 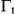 observed to be colonized at time t is
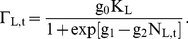
(8a)

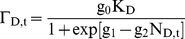
(8b)(see Fig. 4b).

One of the few population models incorporating resources and beetle populations is the resource depletion model by Økland and Bjørnstad [13]. Models of this type assume that when N≤T, K = K_D_, and when N>T, K = K_D_+K_L_, and combine this with a standard population growth model like the Gomperz function.

**Figure 5:**
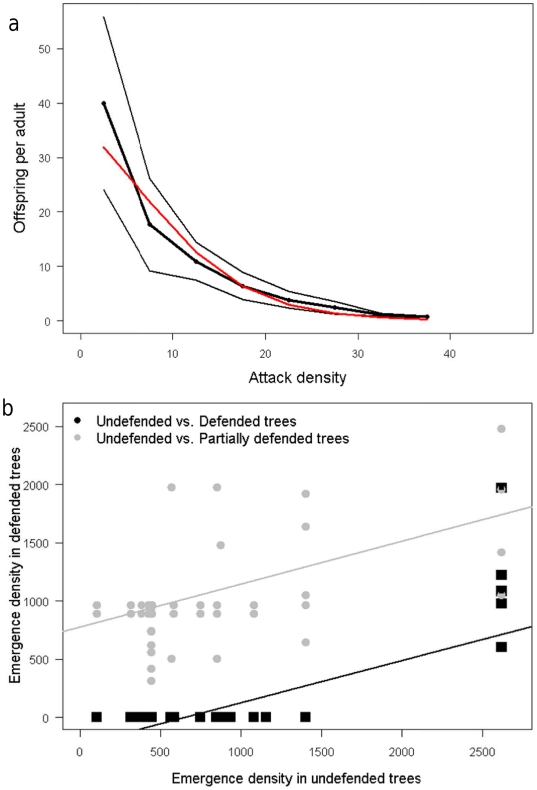
Comparisons with observations. a) Field data on the number of offspring per adult *Ips cembrae* as a function of gallery density (black line with ±2SD), showing a strong negative density-dependence. This is consistent with the model density dependence (red line; part of eq. 3, see below as eq. 8) **b**) The density per m^2^ of newly emerging *I. typographus* for 68 trees with fitted regression lines. Partially defended trees (i.e. trees with partial root contact, grey points) are colonized together with lower dead-wood densities, corresponding to expectations (see Results).

A parameterisation of eq.3, with R = 50, K_D_ = 6, a_1_ = 0,a_2_ = 2, β = 0 and A = N = Attack density of *I.cembrae* is consistent with field data [33] (Fig. 5a)
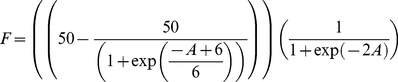
(9)


The SRD obviously involves a number of assumptions and trade-offs between process clarity and realism, being an exploratory model of general use [34]. One key assumption is that beetles can assess their own population density and host availability on “patch” scales seems supported: highly developed olfactory systems allow them to sense different conspecific pheromones, host tree volatiles, and their relative concentrations [3], [4], [7], [22]. On smaller scales other senses may be used [35]. Tree-killing scolytids continuously produce aggregation pheromones until the moment when the host resistance threshold is reached [4], [27]. Manipulating ratios of the predominant monoterpene compound in Norway spruce to *I. typographus* pheromones shows a strong, positive effect on attraction to increasing monoterpene∶pheromone ratios [24], [36]. This is in contrast to *I. pini*, which rarely attack healthy trees and shows a parabolic attraction effect of host monoterpene∶conspecific pheromone ratios [37], intuitively explained as a trade-off between avoiding already densely populated trees and too vigorously defending trees not likely to be successfully colonized.

## Results

The SRD predict that only dead trees will initially be colonized (Fig. 2a), but as the local population density increases, these grow increasingly crowded until density dependence outweighs the risks of either settling in a living tree or emigrating (Fig. 2a–d). The observed dynamics emerge from a single, flexible strategy shared by the whole population [28], but individuals maximizing their fitness does not necessarily imply maximized population growth: the distributions between dead and living trees mostly result in population growth rates far from maximum (Fig. 2c). At low and very high population densities, the SRD and IFD converge, but for a range of population densities when living trees are being colonized, they diverge considerably (Fig. 2d).

### Population dynamics

There is a considerable volume of parameter space where aggregative attacks may occur (Fig. 3 summarizes model behaviour for such a case), and in a subset of these the population growth function gives three non-zero population equilibrium points. The population trajectories can thus be rather complex, and have two attractor basins; one lower (“endemic”) and one higher (“epidemic”) (Fig. 3a). The position of the attractor basins are found to depend on the expected payoff from migration (ω,γ), the risks incurred by initiating an attack (c_L_), the beetles having a substantial effect on the trees (c_0_), the degree to which beetles settling in dead trees first sample random live trees (τ), and of course the abundance of dead (K_D_) and living trees (K_L_) that are not too vigorously defended (T_L_) (see Analysis section).

The population can shift between the endemic and epidemic attractor basins (Fig. 3) by several mechanisms. An epidemic state can be triggered by increased population density, such as following an increased abundance of dead hosts (K_D_), immigration, or increased survival rates. It can also be triggered by drought stress, or increased aggressiveness (in the beetles or the composition of their host-pathogenic fungi), both in effect lowering the colonization threshold (T_L_), increasingly explorative search patterns (increased τ), or even spontaneously when the dynamics are unstable. Even increased environmental variability alone can increase the odds that a population will enter the epidemic attractor basin within a given time period.

Likewise, epidemics may cease from density-dependent offspring reduction, emigration, poor survival (low R), lack of resources (low K), abundant rainfall (increased T_L_) or decreased aggressiveness or exploration (increased T or decreased τ). Another factor shaping dynamic structure is whether early-arriving individuals are able to monopolize resources, thus making them less affected by increasing density than latecomers (Fig. 2b) and stabilizing the dynamics around an equilibrium point.

Low-threshold systems where few beetles are needed to kill moderately healthy hosts can appear stable and endemic as long as beetle populations are low (for instance due to winter mortality, predation and low host abundance), but can easily switch to stable epidemic (i.e., potentially causing large outbreaks) when populations increase (for instance due to increased survival or decreased predation). However, when defence thresholds are very high, living trees are colonized only at densities where reproduction is severely depressed by density dependence and migration mortality, and the population tends to decline rapidly and return to the endemic cycle.

### Evolutionary dynamics

The Ideal Free Distribution (IFD) is a central theoretical concept in ecology, behavioral ecology and evolutionary biology. The term was first coined by Fretwell and Lucas in 1970 to 1971, and has been of central importance in theory development and studies for a range of ecological and evolutionary systems [14], [15], [38], [39]. It describes the way in which animals distribute themselves among resource patches, stating that individual animals will aggregate proportionately to the amount of resources available in each patch. So for instance, if patch A contains twice as much food as patch B, there will be twice as many individuals foraging in patch A as in patch B. The relationship with evolutionary game theory is that when an IFD is achieved, no individual can do better by changing patch. Simple IFDs are rarely observed in nature, as imperfect information, unequal competitive abilities, time lags and costs of redistribution can inhibit the formation of an IFD. However, it is a very useful null assumption, because systematic deviations from it alerts us to the existence of costs and/or constrains that need to be accounted for, or to search for a cause of maladaptive behavior.

The density at which living trees are settled is determined by the beetles' evolved “expectations” of density dependence, but also migration mortality and probability of successfully colonizing living trees. Thus, the patchy and unpredictable distribution of dead trees is a prerequisite for the risky colonization strategy to arise.

As long as redistribution is penalized, there is a considerable population density interval over which individuals settling in living trees, despite the risks from tree defences, have higher expected reproductive output than those in dead trees (Fig. 2d), thus deviating from the IFD. However, selection for aggressiveness is decreased at very high densities, and reversed at very low densities (Fig. 2d), and as all populations exhausting their local host base quicker than it replenishes sooner or later are likely to return to low population densities, selection is highly unlikely to stay directional. As the selective pressure changes depending on the time scale of population fluctuations, this suggests a fitness premium on rapid trait selection such as maternal or epigenetic effects. Such rapid responses to selection would materialize as heterogeneities in aggressive behaviour between otherwise genetically homogenous populations. (It also suggests that if some species could redistribute so as to follow the IFD, this would be antagonistic to the evolution of aggressive strategies, as the best a beetle could hope for when initiating a risky attack on a healthy host would be to break even with its conspecifics in the same patch.)

The “random sampling coefficient” τ represents a little explored effect of beetle behaviour. It denotes the proportion of beetles in a patch that will sample one or more trees by burrowing, and thus contribute to the depletion of tree defences, before they decide to settle in a dead tree. The density threshold at which colonization occurs increases markedly with low values of τ (Fig. 4a), suggesting that species with finely grained host localisation will have fewer outbreaks.

Simulations of the model suggests that there is selection for different levels of “aggressiveness” depending on population density (N) relative to defence threshold (T) and landscape connectivity/patch abundance (Ω).

### Comparing with observations

As predicted by the SRD, *Ips typographus'* reproductive success has been found [40] to be consistently higher in trees colonized while still living than in dead trees at the same sites, despite a clear general preference (i.e., when sufficient amounts are available for both substrates) for dead trees [41].

Experiments suggest that host tree volatiles attract beetles on a “patch” scale, and that most trees are visited and assessed [42] by beetles landing randomly on a finer spatial scale [30]. Using close range chemical signals and/or penetrating into the phloem to assess host suitability, they simply abort the attempt if the tree is either too strongly defended or crowded [25], [35], implying that τ>0 for some aggressive species.

Fat content is negatively affected both by activity (migration) and density [7], [22], and less fat means less chance of surviving to find another host. The SRD model implies that an adaptive reaction norm to signs of increasing migration risk include a higher propensity for attacking trees. Thus, beetles emerging from crowded, brood trees should be more likely to start an infestation close to their parental tree instead of migrating, both due to their density and to their lower energy reserves. This seems to be supported by multiple observations [5], [7], [43], [44].

The colonization sequence observed by by Grégoire et al. [32] were easily reproduced and predicted by the SRD model (see Fig. 4b). Best fit was found when assuming increasing migration risk as the season progresses (ω_1_ = −2, ω_2_ = 0.05), density dependence is moderately steep (c_0_ = 0.2, α = 2), some random sampling from early settlers occur (τ = 0.5), and a medium-range tree defence (T_L_ = 15).

Field data on the number of offspring per adult *I. cembrae* as a function of gallery density suggest a steep density-dependence for this species. If about 20% of the offspring survive to breed, populations will decrease for all but the lowest recorded attack densities [33]. This indicates that density dependence can be a local demographic force, without which the SRD predicts that no beetle would take the risk of initiating an attack on a living tree, and the shape is consistent with the SRD general function choice (Fig. 5a).

The density per m^2^ of newly emerging *I. typographus* in 68 trees was recorded at six sites over two years in the Vosges mountains of France ( Grégoire unpublished data). The living trees were colonized only when the dead trees nearby were colonized at high density, as measured by emergence (Fig. 5b). Living trees with decreased defences (low T) (i.e., trees with partial root contact) follow the expected pattern, being colonized at lower levels of crowding in nearby dead trees.

**Figure 6:**
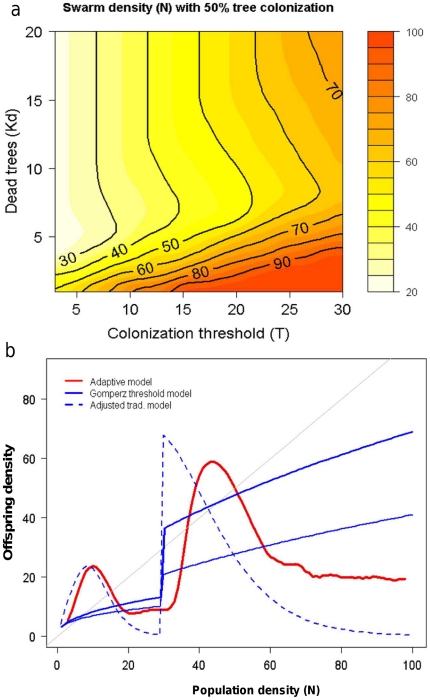
Comparisons with other model formulations. a) Comparing the offspring density for the model (red) with an established resource-based bark beetle model by Økland and Bjørnstad (2006), see Analysis section (blue lines, upper line scaled for better fit). The dotted blue line is the population model presented here, but with the simplification that living trees are colonised immediately when N>T, as is implicit in most current population models that do not take adaptive behaviour into account. **b**) Swarm density for which colonizing living trees has a 50% chance of success, as a function of colonization threshold (T) and dead trees present (K_d_). The interaction is strongly non-linear.

The assumption implicit in some population models that beetles colonize trees to produce new resources when possible (i.e., whenever N>T) creates considerable discrepancies with the SRD, both with respect to densities at which a population can enter an epidemic phase, and to how stable the epidemic can be (Fig. 6).

The beetle density at which tree mortality will occur responds in a strongly non-linear fashion to the interaction between the colonization threshold and the amount of dead trees present. This may be one of the reasons why classical risk and population models have shown relative rather than absolute predictive capability [17] (Fig. 6).

## Discussion

The SRD model supports and provides mechanistic underpinnings for the proposed bimodal population growth curve of aggressive bark beetles [5], [13], [18], [45]. The bimodal density dependence suggests that zones of moderate beetle density may attract individuals from surrounding areas, facilitating waves of attack emanating from outbreak patches. A good example may be the *D. ponderosae* on the Chilcotin Plateau, where strong spatial and temporal dependencies at small scales (<18 km), indicate feedback from local epidemic processes [46].

The beetles may position galleries so as to minimize interference from later arrivals [44], and the larvae develop quickly. But larvae also have a limited ability to cross consumed phloem [47], and the net outcome seems to be a strong decrease in reproductive success with increasing gallery densities [6], [33], [41], [48], [49], [50], [51]. Whatever the consequences for the residents, late-arriving beetles will be selected for entering a crowded bole. The exception would be under strong kin selection, which is unlikely in widely dispersing species [19], [21], [43]. We see that simply dividing the number of beetles on the available resources may give wrong estimates of population growth and stability properties, as the population distributions may not follow an IFD or maximize tree colonization.

The exact host defence threshold (T) only matters when it is higher than the beetle density at which crowding occur in dead trees, meaning that there is no fixed defence threshold below which trees become at risk (complicating predictive risk models [17]). Thus, the gallery densities found in colonized trees are not just a reflection of how many beetles it takes to kill a tree, but the risk of initiating a colonization attempt in a living tree. This is in accordance with the observation that even healthy hosts can be successfully colonized if pheromone-baited [18].

When the SRD and IFD deviate, the “random landing” on a stand scale observed for several species [30] may be adaptive: beetles sampling living trees may increase their fitness (Fig. 2b) by being more likely to become part of an early-stage attack on a living tree than beetles that steer directly to the available dead trees. The exploratory landing pattern is therefore in a feedback relationship with aggressiveness, as less aggressive species will have less to gain by exploratory sampling of living hosts, which again makes colonization less likely and selects for more precise localisation of dead hosts etc. Landing and search patterns are thus predicted to also shape larger-scale spatial dynamics: if the beetles do not sample living hosts to assess defence levels and quality, and are “invisible” to each other while flying (i.e., not emitting pheromones), no living trees will be colonized without sufficient dead hosts nearby to inform beetles about high conspecific densities. Thus landing patterns determine to what degree epidemics can start at random locations and spread efficiently through healthy forests.

The deviation from the IDF may point to a feedback process facilitating the evolution of aggressiveness: as sex pheromones easily lead to beetles aggregating in considerable numbers there is a fitness gain from settling in a living but possibly succumbing tree at the right moment. However, the expected fitness differences converge when dead wood is scarce and beetle densities are high (Fig. 2d), and the selection pressure is reversed when resource depletion, density dependence or reduced survival (cold winters, predation, etc.) depresses the population below the threshold where successful colonization is likely. Thus, “aggressiveness” in bark beetles and their associated fungi seem likely to exist in a state of evolutionary flux or cycles. As heritability on the traits shaping aggressiveness might be mediated through maternal [52] and epigenetic [53] effects as well as allele differences, this may explain evidence for multiple strategies and strongly heritable differences between individuals and populations of the same species when it comes to aggressiveness [5], [18], [41], [54], [55].

Also, the similarities in selective forces and starting points suggest that aggressive life strategies may easily have evolved independently from non-aggressive ancestors in different genera such as *Ips* and *Dendroctonus*.

If beetles continue to be aware of the rest of the patch after starting to burrow into a host, and can relocate without fitness costs, they will distribute proportionally between dead and live trees, and an IFD state will be reached. However, once gallery construction has begun, this is not likely to be the case. Beetles may re-emerge to start a second brood after some time, but for the purposes of this model, any re-emerged individuals are treated as new arrivals and fitness is calculated per brood. The possibility of re-emergence is an incentive to breed as soon as possible. For some individuals, it may still be adaptive to “wait and see” instead of being the first to attack, and the complex pheromone systems of many bark beetles show signs of individual variation [42]. But time is limited, and the beetles with the least fat reserves must commit first, enforcing the sequential individual decisions driving the SRD.

While increasing migration range generally may increase the range of spatial synchrony, this may not be the case under chaotic or strongly resource-driven population dynamics, with consequences for adaptive dispersal patterns. Everything else being equal, species with a lower cost of migration are less prone to attack living trees. But when they do, the epidemics may be more prolonged, as individuals can migrate away rather than cause population crashes. Similarly, if early settlers can monopolize gallery space, density dependence will rarely cause populations to crash.

Integrating data and processes on different scales is difficult, but these results show the need to examine population models for implicit evolutionary assumptions, and doing so generates hypotheses that may explain otherwise puzzling aspects of the species' ecology. In general, the inherent bistability and strong endogenous feedbacks of bark beetle systems makes them particularly sensitive to perturbations such as climate change [3], and adaptations to rapidly fluctuating selective pressures [52] may allow species to respond quickly to ecological changes such as decreased host defences following changes in climate or forestry.
